# Screening for Depression in Pregnant Women with Fetal Anomalies: A Pilot Study

**DOI:** 10.1192/j.eurpsy.2026.12043

**Published:** 2026-07-31

**Authors:** M. Marković, M. Gojković, D. Mladenović, I. Jovanović, S. Kostić, M. Petronijević, S. Vrzić Petronijević

**Affiliations:** 1 Clinic for Psychiatry, University Clinical Centre of Serbia, Belgrade; 2 Special Hospital for Psychiatric Disorders “Kovin”, Kovin; 3 Clinic for Gynecology and Obstetrics, University Clinical Centre of Serbia; 4 School of Medicine, University of Belgrade, Belgrade, Serbia

## Abstract

**Introduction:**

The diagnosis of a fetal anomaly and the subsequent decision to terminate pregnancy may serve as precipitating factors for psychological distress in the expectant mother. Understanding the psychological impact of fetal anomalies is crucial for tailoring targeted interventions in this vulnerable population, where two sources of vulnerability—pregnancy itself and the presence of a fetal anomaly—are intertwined. There is a lack of studies monitoring the mental health of these women.

**Objectives:**

The aim of this study was to examine the presence of psychopathological phenomena and the influence of various sociodemographic and gynecological–obstetric factors on their occurrence in pregnant women with fetal anomalies.

**Methods:**

This was a prospective pilot study that recruited pregnant women with fetal anomailes. All participants completed the Edinburgh Postnatal Depression Scale (EPDS) for screening depressive symptoms, while sociodemographic, gynecological–obstetric, and psychiatric variables were systematically recorded.

**Results:**

A total of 16 pregnant women with fetal anomalies were included (mean age 30.6 years, range 21–46). Screening with the EPDS yielded a mean score of 9.25 (range 1–22), with 56% (n=9) scoring above the cut-off of 10, indicating clinically relevant depressive symptoms. Most participants rated their relationship with a partner as highly satisfactory (mean 4.69/5), with no significant correlation with EPDS scores (ρ = –0.309, p = 0.245). Marital status (62.5% married vs. 37.5% cohabiting) and number of children were not significantly associated with depressive symptoms (p > 0.4). Previous miscarriages (25% natural, 18.7% medical) showed no significant association (p = 1.0). Education, employment status, financial satisfaction, and place of residence were also not significantly related to EPDS scores (all p > 0.2).

**Conclusions:**

More than half of the pregnant women with fetal anomalies in this pilot study screened positive for clinically relevant depressive symptoms, highlighting their psychological vulnerability. However, no significant associations were found between depressive symptoms and sociodemographic or obstetric variables, likely reflecting the small sample size. These findings are showing that there is the need to integrate mental health assessment into clinical care pathways for pregnancies complicated by fetal anomalies. There is a need for larger, prospective studies to clarify risk factors and inform targeted interventions.

**Disclosure of Interest:**

None Declared

